# An Independent Prognostic Model Based on Ten Autophagy-Related Long Noncoding RNAs in Pancreatic Cancer Patients

**DOI:** 10.1155/2022/3895396

**Published:** 2022-05-14

**Authors:** Jiahui Tian, Chunyan Fu, Xuan Zeng, Xiaoxiao Fan, Yi Wu

**Affiliations:** ^1^Department of Laboratory, The First Affiliated Hospital of Hunan Normal University, Changsha, Hunan 410005, China; ^2^Department of Medicine, Hunan Normal University, Changsha, Hunan 410005, China

## Abstract

**Purpose:**

Pancreatic cancer (PC) is a common, highly lethal cancer with a low survival rate. Autophagy is involved in the occurrence and progression of PC. This study aims to explore the feasibility of using an autophagy-related long noncoding RNA (lncRNA) signature for assessing PC patient survival.

**Methods:**

We obtained RNA sequencing and clinical data of patients from the TCGA website. Autophagy genes were obtained from the Human Autophagy Database. The prognostic model, generated through univariate and multivariate Cox regression analyses, included 10 autophagy-related lncRNAs. Receiver operating characteristic (ROC) curves and forest plots were generated for univariate and multivariate Cox regression analyses, to examine the predictive feasibility of the risk model. Gene set enrichment analysis (GSEA) was used to screen enriched gene sets.

**Results:**

Twenty-eight autophagy-related lncRNAs were filtered out through univariate Cox regression analysis (*P* < 0.001). Ten autophagy-related lncRNAs, including 4 poor prognosis factors and 6 beneficial prognosis factors, were further screened via multivariate Cox regression analysis. The AUC value of the ROC curve was 0.815. GSEA results demonstrated that cancer-related gene sets were significantly enriched.

**Conclusion:**

A signature based on ten autophagy-related lncRNAs was identified. This signature could be potentially used for evaluating clinical prognosis and might be used for targeted therapy against PC.

## 1. Introduction

Pancreatic cancer (PC) is a highly aggressive cancer that affects the digestive system. It has an incidence rate of 8% in 5 years and is the 11^th^ most common malignant tumor [1, 2]. It is difficult to diagnose PC at an early stage, owing to its insidious onset [3]. Currently, surgery is the only effective treatment available for patients with pancreatic cancer. However, pancreatic tumors are often characterized by a poor patient prognosis due to delayed diagnosis [4, 5]. Although notable advances in the use of these technologies for PC screening and testing have been achieved, certain limitations are associated with the complete eradication of tumors. It is imperative to explore potent therapeutic targets and emerging prognostic biomarkers for the improved diagnosis and treatment of PC [6]. Autophagy is a cellular catabolic mechanism that facilitates the maintenance of cellular homeostasis through the degradation of cytoplasmic metabolites [7, 8]. Autophagy is involved in the progression of many cancers and may prevent or enhance tumor growth, depending on the tumor type and stage [9, 10]. Though autophagy may protect cells from harmful substances in the early stage of tumorigenesis, the adaptive metabolic response generated during autophagy results in nutritional conditions for tumor progression under an unfavorable environment [11]. Recent studies have focused on applications involving the use of autophagy genes for targeted anticancer therapy [12].

Long noncoding RNAs (lncRNAs) are transcripts with a length of more than 200 nucleotides that lack protein-coding functions. LncRNAs are involved in important biological processes, such as proliferation, differentiation, apoptosis, invasion, and metastasis, in patients with cancer [13, 14]. Recently, increasing levels of evidence indicate that lncRNAs are primarily involved in the occurrence and development of cancer, and this might also be related to their potential role in regulating autophagy [15, 16]. Sun et al. determined the prognostic signature based on autophagy-related lncRNAs (ATRlncRNAs); this signature might potentially be used for targeted therapy and evaluation of the prognosis of colon cancer patients [17, 18]. Liu et al. investigated the clinical role of ATRlncRNAs in cholangiocarcinoma and demonstrated its potential for the personalization of cholangiocarcinoma treatment [19]. Deng et al. proposed a six-ATRlncRNA signature that might be helpful for individualized therapy and PC patient assessment. Furthermore, LINC01559 may play a critical role in alleviating the resistance to gemcitabine [20]. Feng et al. demonstrated the correlation between ATRlncRNAs and many cancers, including cervical cancer, breast cancer, glioma, and lung adenocarcinoma [21–24]. Thus, the use of ATRlncRNAs as prognostic biomarkers for PC patients may provide new indicators for the early diagnosis and prognostic judgment of PC patients.

We comprehensively analyzed the correlation between RNA sequencing (RNA-seq) data and autophagy genes. Subsequently, we developed a prognostic signature based on ten ATRlncRNAs that were found in PC patients. Numerous biographical analysis results demonstrated that the ATRlncRNA signature could be extensively used in prospective applications, for molecular diagnosis, prognostic evaluation, and targeted treatment of PC patients.

## 2. Materials and Methods

### 2.1. Collection of Datasets and Clinical Information of PC Patients

The RNA-seq and clinical data of PC patients were acquired from the TCGA website (https://portal.gdc.cancer.gov/). Details regarding futime, fustat, age, sex, grade, stage, and tumor-node-metastasis classification were included in the clinical information. Incomplete clinical information was removed.

### 2.2. Selection of Autophagy-Related Genes of PC Patients

The autophagy genes (ATGs) were obtained from the Human Autophagy Database (https://www.autophagy.lu/index.html). The lncRNA expression levels were normalized by log2 transformation, using the edgeR package. Pearson's correlation test was used for the screening of lncRNAs that were related to autophagy genes. The criteria used to identify the ATRlncRNAs were as follows: correlation coefficient |*R*^2^| > 0.4 and *P* < 0.001. Cytoscape software (version 3.8.2) was used to construct coexpression networks.

### 2.3. Construction of the Prognostic Model

Univariate Cox regression analysis was used to identify prognostic ATRlncRNAs (*P* < 0.001). Then, multivariate Cox regression analysis was performed to optimize the risk model, and ten ATRlncRNAs were included in the formula to assess PC patient prognosis.

(1) $$\begin{array}{c} \text{Risk score}=\sum \beta *\left(\text{expression of IncRNAs}\right), \end{array}$$

where “*β*” is the regression coefficient for each gene.

Patients were split into two (low-risk and high-risk) subgroups, based on the median risk score. Kaplan-Meier survival curve analysis was applied to compare the differences in survival between these subgroups.

### 2.4. Feasibility of the Risk Model for Clinical Evaluation

First, to examine the independent predictive ability of the risk score and clinical factors, we constructed forest plots for univariate and multivariate Cox regression analyses. Subsequently, the receiver operating characteristic (ROC) curve was applied, to detect the validity of the risk assessment system, via a comparison with the area under the curve (AUC).

### 2.5. Functional Analysis

Gene set enrichment analysis (GSEA, https://www.broadinstitute.org/gsea/index.jsp) was used to screen deferentially expressed genes. It was used to analyze whether gene sets between the high and low expression groups differed during the process of autophagy.

## 3. Results

### 3.1. Construction of a Coexpression Network for ATRlncRNAs

A total of 182 lncRNAs were downloaded from TCGA, and a total of 232 autophagy-related genes were downloaded from the Human Autophagy Database (HADb, https://www.autophagy.lu/). Consequently, 176 PC patients and their complete clinicopathological information were sifted out for subsequent analysis.

### 3.2. Establishment of a Prognostic Model on ATRlncRNAs

**Figure 1:**
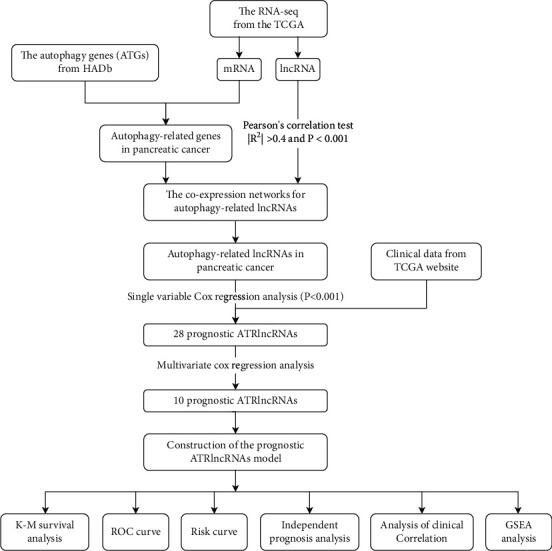
The workflow of this study.

The workflow revealed the detailed work of this study (Figure 1). The 28 prognostic ATRlncRNAs used for assessing survival were filtered using univariate Cox regression analysis (*P* < 0.001, Table 1). Besides, we further screened 10 prognostic ATRlncRNAs on the basis of the above 28 autophagy lncRNAs via multivariate Cox analysis. Of these, 4 lncRNAs were poor prognosis factors (AC245041.2, AC036176.1, LINC01089, and LINC02257) and 6 lncRNAs were beneficial prognosis factors (FLVCR1-DT, AC006504.7, AC125494.2, AC012306.2, ST20-AS1, and AC005696.1) (Table 2).

**Figure 2:**
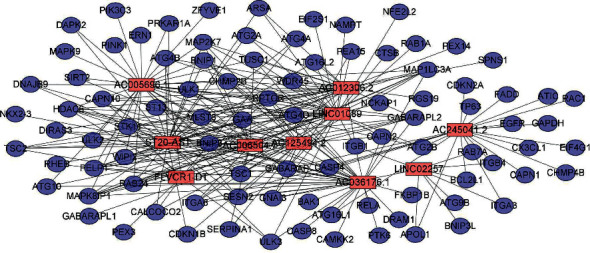
Coexpression network for autophagy-related genes and ten independently diagnosed lncRNAs. The red rectangular nodes represent independently diagnosed lncRNAs, and the blue and purple round nodes represent autophagy-related genes.

**Figure 3:**
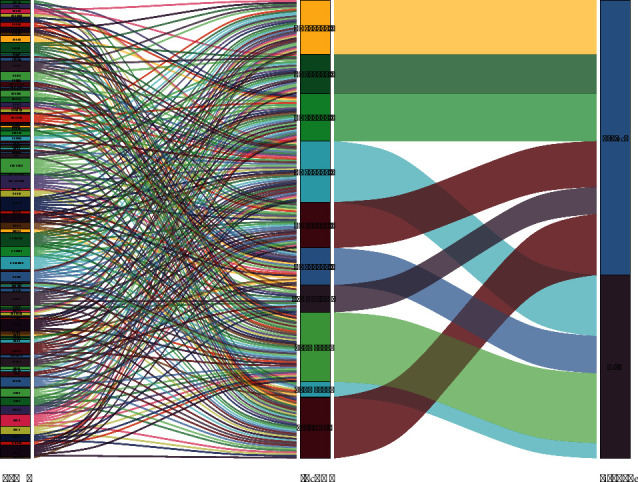
The relationship between autophagy-related genes, independently diagnosed lncRNAs, and risk types was demonstrated in the Sankey diagram.

**Figure 4:**
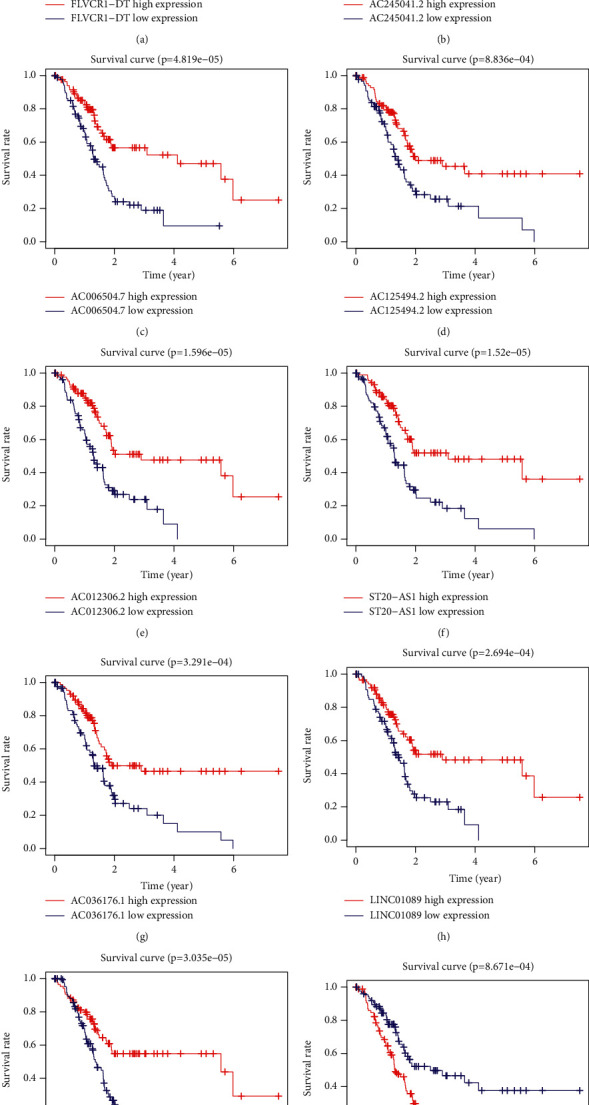
Kaplan–Meier survival curve of nine independently diagnosed lncRNAs in PC. Four lncRNAs were poor prognosis factors (AC245041.2 (B), AC036176.1 (G), LINC01089 (H), and LINC02257 (J)). Six lncRNAs were beneficial prognosis factors (FLVCR1-DT (A), AC006504.7 (C), AC125494.2 (D), AC012306.2 (E), ST20-AS1 (F), and AC005696.1 (I)).

Accordingly, we established a coexpression network for the 10 ATRlncRNAs, to determine the interactions between the autophagy genes and prognosis-related lncRNAs (Figure 2). Based on the results shown in the Sankey diagram, the association between autophagy-related genes, prognosis-related lncRNAs, and related risk types has been derived, as shown in Figure 3. The Kaplan–Meier survival curve further indicated that the 10 ATRlncRNAs were closely related to a prognosis of PC (*P* < 0.001, Figures 4(a)–4(j)).

### 3.3. Evaluation of the Prognostic ATRlncRNA Model

**Figure 5:**
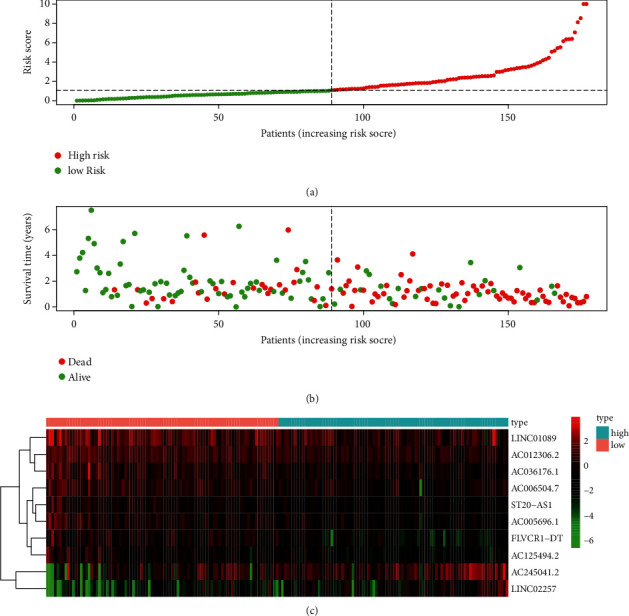
Risk score evaluation of a prognostic, autophagy-related lncRNA model of PC. (a) Risk score of the risk model. (b) The scatter plot reflects the survival period of PC patients. (c) Heat map demonstrating 10 differentially expressed prognostic lncRNAs is exhibited.

**Figure 6:**
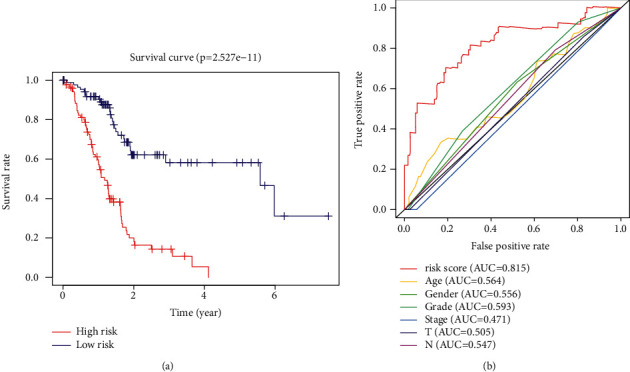
Prognostic impact of the risk signature. (a) Kaplan–Meier survival curve of the risk model. (b) Risk score of the ROC curve and other clinical factors based on the AUC. AUC, acute area under the curve.

A risk model of prognostic ATRlncRNAs was established based on the risk score. Patients were divided into two groups, including the high-risk and low-risk groups. Patients with a higher overall survival (OS) in the low-risk group were better illustrated through the risk curve and scatter plot (*P* < 0.001) (Figures 5(a) and 5(b)). The construction of a heat map enabled the visualization of 10 differentially expressed prognostic lncRNAs, as shown in Figure 5(c). Furthermore, KM (Kaplan–Meier) survival analysis showed that the low-risk group had a better prognostic impact than the high-risk group (*P*=2.527*e* − 11, Figure 6(a)). The ROC curve demonstrated in Figure 6(b) was used to evaluate the diagnostic value of the risk model. The AUC value for ATRlncRNAs was 0.815, which showed that the risk model exhibited potential for the evaluation of the prognosis of PC.

### 3.4. Correlation Analysis of Clinical Characteristics and Risk Models of PC

**Figure 7:**
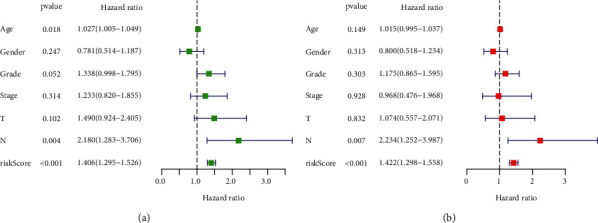
Predictive performance evaluation of the risk model based on the risk score and clinical factors using the forest plots for univariate (a) and multivariate (b) Cox regression.

To determine whether the risk model was an independent prognostic factor for PC survival analysis, univariate and multivariate Cox regression analyses were performed, as shown in Figures 7(a) and 7(b). The results of both these analyses revealed that the risk score could act as an effective prognostic factor (univariate regression: HR = 1.406, 95% CI = 1.295–1.526, *P* < 0.001; multivariate regression: HR = 1.422, 95% CI = 1.298–1.558, *P* < 0.001). Details regarding clinical factors, including age, sex, stage, and tumor-node-metastasis status, have been shown in Table 3. A significant difference was observed in the risk score.

### 3.5. Gene Set Enrichment Analysis

**Figure 8:**
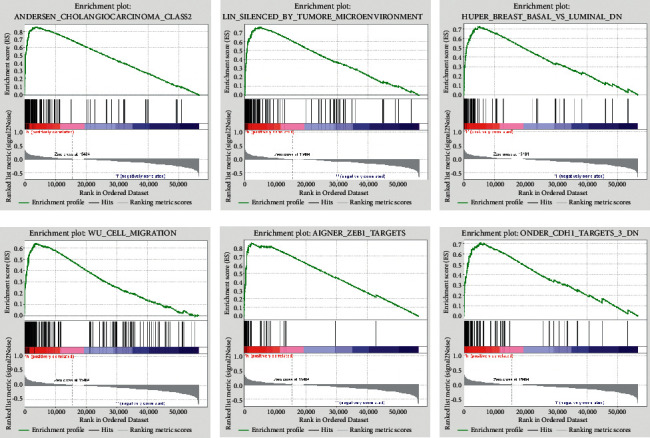
GSEA analysis of deferentially expressed genes.

Based on GSEA results, deferentially expressed genes were screened out. Seven lncRNAs were upregulated in high-risk groups at an FDR <0.05 and a nominal *P* value <0.01. Several sets, including those for cell migration, ZEB1 targets, EGFR signaling, lin genes silenced by the tumor microenvironment, and CDH1 targets (Figure 8), were all closely linked to cancer. These results give rise to the possibility that the diagnosis and treatment of PC could be achieved using this method.

## 4. Discussion

Pancreatic cancer (PC) remains one of the most aggressive cancers worldwide. The mortality rate of PC tends to be similar to its morbidity rate despite the development of advanced treatment techniques [25, 26]. Recent studies have mainly focused on the use of biomarkers, such as lncRNAs, for targeted therapies in PC; this may drive the ability to provide customized treatment and optimize the therapeutic effects [27–29]. Autophagy has positive and negative regulatory effects on PC, based on the setting and stage [30]. Emerging evidence has proven that lncRNAs may serve as prognostic and diagnostic biomarkers during the initiation and development of PC [29, 31]. Lou et al. discovered that lncRNA HULC participates in the Wnt/*β*-catenin signaling pathway in pancreatic cancer cells and could be considered as an effective biomarker for the diagnosis of PC [32]. However, there have been few reports on the selection of autophagy-related lncRNAs for the assessment of PC prognosis. Consequently, we developed a risk model for evaluating the survival of PC patients through the screening of autophagy-related lncRNAs.

Based on the formed coexpression network, we selected autophagy-related lncRNAs for assessing survival in PC patients. Second, 10 autophagy-related lncRNAs, including AC245041.2, AC036176.1, LINC01089, LINC02257, FLVCR1-DT, AC006504.7, AC125494.2, AC012306.2, ST20-AS1, and AC005696.1 were obtained through Cox regression analysis. Future studies need to assess whether these 10 risk factors can be used for prognostic assessment of PC.

Eight autophagy-related lncRNAs, including AC245041.2, AC036176.1, LINC01089, LINC02257, FLVCR1-DT, AC125494.2, AC012306.2, and ST20-AS1, were found to be involved in cancer prognosis. There have been no detailed reports on the role of AC006504.7 and AC005696 in cancer risk assessment to date. (1) The lncRNA AC245041.2 and mRNA LAMA3, which are strongly correlated with each other, are relevant to the detection of KRAS mutations, which may be indicative of a poor prognosis of PC [33]. Cao et al. screened out multiple autophagy-associated lncRNAs, including AC245041.2, based on bioinformatic analyses, to develop a risk model for the prognostic analysis of PC [20]. ApoL1 is one of the interacting autophagy-related genes that express AC245041.2. When ApoL1 was overexpressed intracellularly, it could induce autophagy and autophagy-associated cell death in all cell types. In contrast, when the ratio of ApoL6 : ApoL1 was elevated, it could promote apoptosis via the inhibition of autophagic signals [34]. (2) AC036176.1, a ferroptosis-related lncRNA, may act as a prognostic biomarker for PC [35]. ATG16L1 is one of the interacting ATGs of AC036176.1; the stimulation of *Mir223* could downregulate the levels of ATG16L1 below the threshold level, therefore resulting in the inhibition of autophagic activity. This establishes ATG16L1 as an important target of *Mir223*, which is closely involved in the autophagy process [36]. (3) LINC01089 was proven to serve as an influential factor that affected cancer development [37]. (4) LINC02257 was expressed at high levels in colorectal cancer patients and seemed to act as a hazard factor [38]. Xu et al. noted that LINC02257 and FLVCR1-DT were utilized as an adverse lncRNA and favorable lncRNA, respectively, for monitoring the model-based prognosis prediction of PC patients [39]. RPTOR is one of the ATGs interacting with LINC02257; a study found that the expression of miR-377-3p could target RPTOR and induce autophagy in vivo and in vitro [40]. (5) AC125494.2 proteins were also reported to act as factors that were beneficial for assessing PC patient survival [20]. (6) Shen et al. predicted that AC012306.2 is positively correlated with the occurrence of cervical cancer [41]. (7) The immune-related lncRNA ST20-AS1 model was established in patients with anaplastic gliomas [42].

Ten autophagy-related lncRNAs were filtered to develop a risk signature for prognostic identification in PC patients. The results of univariate and multivariate Cox analyses further illustrated their reliability and demonstrated that the ten autophagy-related lncRNAs acted as prognostic factors that could predict overall survival in the model. Subsequently, on the basis of the coexpression network and Sankey diagram, the relationship between the mRNAs and lncRNAs was visualized. The AUC value was 0.815, which illustrated the feasibility of using the model for predicting the prognosis of PC patients.

GSEA analysis suggested that the gene set was primarily centered on pathways associated with tumor progression, metastasis, and cell migration. For example, in the two crucial gene sets Zeb1 and EGFR, Zeb1 is identified as one of the key EMT genes, and its overexpression is linked to tumor metastasis [43]. EGFR has received extensive attention from researchers focused on the field of targeted anticancer therapy. The downregulation of EGFR signaling via CDF compounds upregulating miR-146a might provide a new therapeutic option against PC [44]. EGFR is associated with resistance to conventional cancer therapy; resistance to EGFR-targeted therapy can be attenuated via autophagy inhibition and thus represent a new mode of tumor treatment [45].

Certain limitations are associated with this study. (1) The database is a single-source database and the study data are limited to 182 cases; the documentation of the clinical features is also incomplete. (2) Additional validation studies, including those involving an independent cohort, need to be conducted, to verify the potential of the prognostic model for the purpose of making assessments. (3) In this study, only data analysis was performed; the explicit function of autophagy-related lncRNAs in disease prognosis was not validated by performing relevant experiments.

In conclusion, ten autophagy-related lncRNAs were used to determine a prognostic signature for PC patients. The prognostic factors in the signature may facilitate the development of novel methods for targeted therapy and clinical evaluation of PC.

## Figures and Tables

**Table 1:** Univariate Cox analysis showed that 28 autophagy-related lncRNAs were significantly related to the survival of PC patients.

Gene	KM	B	SE	HR	HR. 95 L	HR. 95 H	*P* value
FLVCR1-DT	<0.001	−1.049	0.273	0.350	0.205	0.598	<0.001
AC064836.2	<0.001	−0.512	0.141	0.599	0.454	0.790	<0.001
LINC01004	<0.001	−0.401	0.105	0.670	0.546	0.823	<0.001
AC245041.2	<0.001	0.201	0.051	1.222	1.106	1.350	<0.001
AC142472.1	<0.001	−0.983	0.265	0.374	0.223	0.629	<0.001
AC006504.7	<0.001	−0.816	0.196	0.442	0.301	0.649	<0.001
AC125494.2	0.001	−1.299	0.327	0.273	0.144	0.518	<0.001
AC012306.2	<0.001	−0.666	0.155	0.514	0.379	0.696	<0.001
ST20-AS1	<0.001	−1.629	0.412	0.196	0.087	0.440	<0.001
PTOV1-AS2	<0.001	−0.196	0.055	0.822	0.739	0.915	<0.001
AC036176.1	<0.001	−0.677	0.191	0.508	0.349	0.739	<0.001
U62317.1	<0.001	0.112	0.033	1.119	1.048	1.194	0.001
AC005332.3	<0.001	−0.307	0.069	0.736	0.643	0.842	<0.001
AC127024.5	<0.001	−0.661	0.156	0.516	0.380	0.701	<0.001
AL513165.1	<0.001	−0.232	0.066	0.793	0.697	0.902	<0.001
AL022328.1	<0.001	−0.556	0.161	0.574	0.418	0.787	0.001
AL358472.2	<0.001	−1.245	0.299	0.288	0.160	0.518	<0.001
LINC01089	<0.001	−0.270	0.075	0.764	0.660	0.884	<0.001
AC005332.6	<0.001	−0.162	0.044	0.851	0.781	0.927	<0.001
AC005696.1	<0.001	−1.048	0.260	0.350	0.211	0.583	<0.001
AL122010.1	0.001	−0.619	0.149	0.538	0.402	0.721	<0.001
AC020765.2	0.001	−0.923	0.276	0.397	0.232	0.682	0.001
LINC02257	0.001	0.423	0.093	1.526	1.271	1.832	<0.001
AC005332.5	<0.001	−0.559	0.151	0.572	0.425	0.769	<0.001
AC090114.2	<0.001	−0.865	0.215	0.421	0.276	0.642	<0.001
LINC01705	0.001	0.103	0.026	1.108	1.053	1.167	<0.001
AC145207.5	<0.001	−1.057	0.269	0.347	0.205	0.589	<0.001
AL022328.4	<0.001	−1.150	0.293	0.317	0.178	0.562	<0.001

**Table 2:** Multivariate Cox regression analysis of 10 lncRNAs.

Id	Coef.	HR
FLVCR1-DT	−0.512	0.599
AC245041.2	0.261	1.298
AC006504.7	−0.568	0.566
AC125494.2	−1.199	0.301
AC012306.2	−0.540	0.583
ST20-AS1	−0.694	0.499
AC036176.1	0.406	1.501
LINC01089	0.258	1.294
AC005696.1	−0.578	0.561
LINC02257	0.277	1.319

**Table 3:** Correlation analysis of clinical characteristics of PC patients and risk models.

Clinical	Group	*n*	Mean	SD	*t*	*P* value
Age	≤5	87	1.674	1.651	−0.553	0.581
	>65	81	1.835	2.081		

Gender	Female	76	1.694	1.787	−0.364	0.716
	Male	92	1.799	1.939		

Grade	G1-2	118	1.614	1.599	−1.265	0.21
	G3-4	50	2.077	2.37		

Stage	Stage I-II	161	1.755	1.89	0.169	0.87
	Stage III-IV	7	1.667	1.317		

*T*	T1-2	28	1.186	1.439	−2.141	0.037
	T3-4	140	1.865	1.926		

*N*	N0	47	1.683	2.274	−0.259	0.797
	N1	121	1.778	1.693		

## Data Availability

The RNA-seq and clinical data can be obtained from the TCGA website; the autophagy genes (ATGs) are available at the Human Autophagy Database. The standardized data used for bioinformatics analysis are included within the supplementary information files.

## Supplementary Materials

Supplementary Materials
*Table S1.* The RNA-seq data from the TCGA website. *Table S2. Clinical* data from the TCGA website. *Table S3.* The core results showed a significant correlation between autophagy-related genes and lncRNAs. *Table S4.* The expression of 28 autophagy-related lncRNAs. *Table S5.* The risk data used for the plotting of the heat map, risk curve, and scatter plot for KM survival analysis.
